# Label-Free Imaging of Lipid Depositions in *C. elegans* Using Third-Harmonic Generation Microscopy

**DOI:** 10.1371/journal.pone.0084431

**Published:** 2014-01-02

**Authors:** George J. Tserevelakis, Evgenia V. Megalou, George Filippidis, Barbara Petanidou, Costas Fotakis, Nektarios Tavernarakis

**Affiliations:** 1 Institute of Electronic Structure and Laser, Foundation for Research and Technology, Crete, Greece; 2 Physics Department, University of Crete, Crete, Greece; 3 Institute of Molecular Biology and Biotechnology, Foundation for Research and Technology, Crete, Greece; 4 Medical School, University of Crete, Crete, Greece; University of North Carolina, United States of America

## Abstract

Elucidation of the molecular mechanisms regulating lipid storage and metabolism is essential for mitigating excess adiposity and obesity, which has been associated with increased prevalence of severe pathological conditions such as cardiovascular disorders and type II diabetes, worldwide. However, imaging fatty acid distribution and dynamics *in vivo*, at the cellular or organismal level is challenging. We developed a label-free method for visualizing lipid depositions *in vivo*, based on third harmonic generation (THG) microscopy. THG imaging requires a single pulsed-laser light source, alleviating the technical challenges of implementing coherent anti-Stokes Raman scattering spectroscopy (CARS) to detect fat stores in living cells. We demonstrate that THG can be used to efficiently and reliably visualize lipid droplets in *Caenorhabditis elegans*. Thus, THG microscopy offers a versatile alternative to fluorescence and dye-based approaches for lipid biology research.

## Introduction

Visualization and monitoring of lipid depositions in living organisms is of critical importance for the study of the molecular mechanisms regulating fatty acid metabolism. Aberrant lipid biogenesis, storage and turnover have been implicated in increasingly prevalent human pathologies such as type II diabetes, obesity, cardiovascular diseases and cancer. However, lipids are molecules of no intrinsic fluorescence, and are not easily accessible to lipophilic dyes, *in vivo*. Currently available dye-based methods require destructive fixation and permeabilization of biological samples to provide solely qualitative information, relevant to lipid content and distribution [1]–[3]. An alternative, label-free approach has been employed in recent years for visualizing fatty acids, based on Coherent Anti-stokes Raman Scattering (CARS) and Stimulated Raman Scattering (SRS) microscopy [2], [4], [5]. These methodologies depend on intrinsic molecular vibration as a source of contrast to image DNA, proteins, lipids and small metabolites, without chemical labeling, *in vivo*. Lipids are preferentially imaged in a semi-quantitative manner by CARS and SRS, due to peak-intensity signals generated by CH_2_, C-C, C = C and C = O, aliphatic chemical groups that are abundant in fatty acids. Despite the relative specificity and label-free imaging they afford, CARS and SRS have not gained widespread adoption for routine visualization of lipid depositions for two main reasons. First the cost of acquiring CARS/SRS microscopy instrumentation is substantial and second, setting up and operation of a CARS/SRS microscope requires considerable expertise in nonlinear optics and attentive maintenance, including frequent fine-tuning and precise alignment of two tightly synchronized, mode-locked, ultra-fast laser light sources.

## Results and Discussion

**Figure 1:**
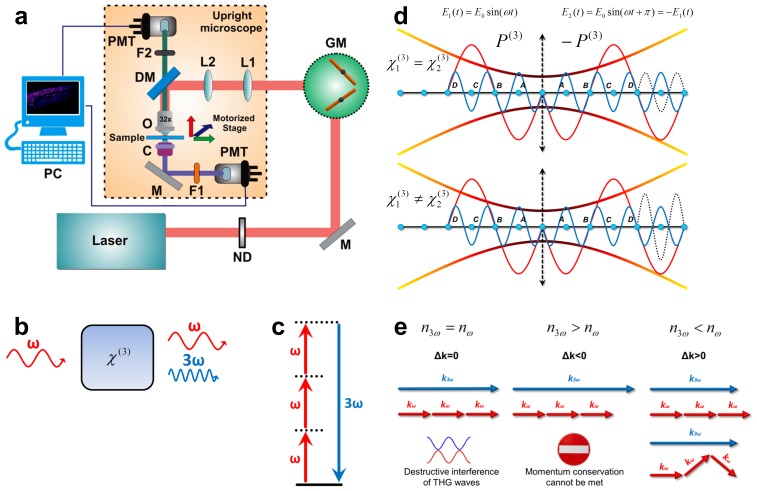
Third harmonic generation microscopy for lipid deposition imaging. (**a**) Schematic representation of the non-linear microscope configuration. Two paths are used for detection, one in reflection (for TPEF signals) and the other in transmission mode (for THG signals). ND: Neutral Density filters, GM: Galvanometric mirrors, PMT: photomultiplier tube, L1, L2: telescope lenses, DM: Dichroic mirror, F1, F2: Filters, O: Objective lens, C: Condenser lens, M: Mirror. (**b**) Geometry of third harmonic generation (fundamental frequency ω), via non-linear optical interaction with a medium (). (**c**) The respective energy-level diagram describing the interaction of three photons of angular frequency ω with a non-linear material, to form one photon of triple frequency (and thus energy) compared to initial incident photons. (**d**) When a Gaussian beam is focused within the volume of a birefringent medium that perfectly compensates dispersion for the fundamental and third harmonic frequencies, THG waves symmetrical to the beam waist position (blue and dotted line respectively) interfere destructively due to a phase shift by π radians, and no signal is obtained. Destructive interference taking place in such a birefringent material can be avoided if the regions before and after the beam waist position possess unequal third order susceptibility values, since the generated third harmonic waves differ in amplitude. (**e**) For a material presenting a perfect dispersion compensation between frequencies ω and 3ω (implying that the phase matching condition Δk = 3k_ω_ - k_3ω_ = 0 is satisfied), the momentum conservation of the respective photons can be met. However, no signal is generated due to destructive interference of THG waves (shown in panel d). For common normal dispersive material (n_3ω_>n_ω_), momentum conservation condition cannot be met in any manner; and therefore, effective THG is not possible. If n_3ω_<n_ω_, efficient THG can occur since the angularly spread wave vectors of the focused fundamental beam can be added effectively so that the total momentum before and after the nonlinear interaction is conserved.

To overcome the limitations of dye-based and CARS/SRS microscopy, we developed a system for label-free imaging of lipids in**vivo, based on Third Harmonic Generation (THG), by modifying a readily available Two-Photon Excited Fluorescence (TPEF) microscope (**Figure 1a**; Materials and Methods). THG is a coherent nonlinear scattering phenomenon, whereby three photons of angular frequency ω are destroyed and a photon of angular frequency 3ω is simultaneously created, in a single quantum mechanical process [6] (**Figure 1b, c**). The THG process is sensitive to local differences in third-order nonlinear susceptibility χ^(3)^, refractive index, and dispersion [7] (**Figure 1d**; Materials and Methods, File S1). In THG microscopy the contrast arises from interfaces and optical heterogeneities of size comparable to the beam focus [8], [9]. No THG signal is collected when the laser beam is focused inside a homogeneous, normally dispersive medium [6], [10] (**Figure 1e**; File S1). Lipid depositions in living cells and tissues have optical properties and nonlinear susceptibility χ^(3)^ values distinct from the surrounding aqueous environment, causing abrupt changes of the refractive index at the interface. Such optical discontinuities are prominent third harmonic generating structures, and provide a source of contrast for preferential imaging of lipids [11].

**Figure 2:**
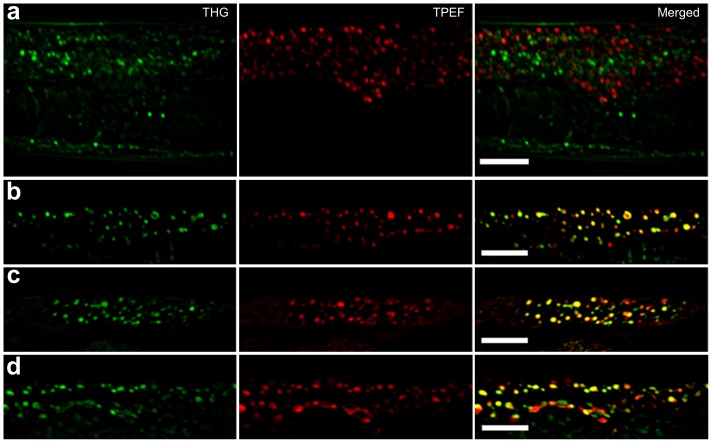
THG specifically visualizes lipid depositions in *C. elegans*. (**a**) Lipofuscin autofluorescence (imaged here by TPEF and pseudocolored in red) does not overlap with THG signals. Images of the mid body region of wild type *C. elegans* are shown (**b**, **c**, **d**) THG signals co-localize with Bodipy 500/510 (in panel b), Nile Red (in panel c), and Oil Red-O (in panel d), labeled material in *C. elegans*, visualized by TPEF. Scale bars denote 20 mm.

To evaluate the sensitivity and specificity of THG microscopy, we imaged lipid depositions in live *C. elegans* animals. Owing to its genetic tractability and transparency, *C. elegans* has become a model organism of choice for the dissection of the molecular mechanisms regulating fatty acid metabolism [12]–[14]. The main fat storage tissue of the nematode is the intestine, and visualization of lipid droplets in intestinal cells has been used as a tool to identify genes controlling fat storage [1], [15]. Both lipophilic dyes and label-free methods based on CARS and SRS have been used to image lipid depositions in *C. elegans* [2], [16]. We combined THG and TPEF microscopy in a single setup to examine nematode intestinal cells. The TPEF modality can be conveniently used, simultaneously with THG, to provide complementary information on fluorescent structures in the tissues imaged. We detected strong THG signals emanating from intestinal cells of live, wild type animals that were not labeled with lipophilic dyes (**Figure 2a**). Structures visualized by THG were clearly distinct from autofluorescent lipofuscin puncta detected by TPEF and conventional single-photon confocal microscopy (**Figure 2a**). THG signals are effectively generated by formations that present optical discontinuities with regard to the linear refractive index and/or third order susceptibility values compared to the surrounding environment. Thus, THG imaging can provide information relevant to the anatomy and tissue morphology of the animal. However, optical discontinuities due to lipid depositions generate significantly more intense THG signals [11], which can be up to an order of magnitude higher, compared to other structures such as membranes and mitochondria, under the same illumination conditions. Therefore, specific visualization of lipid droplets can be achieved by thresholding stacks of obtained images during data processing to eliminate lower intensity THG signals originating from surrounding structures. To further confirm the lipid droplet origin of THG signals, we imaged fixed *C. elegans* animals, stained with Bodipy, Nile Red or Oil Red-O, three fluorescent dyes routinely used to visualize lipid depositions in *C. elegans* [2], [17]. Dye fluorescence was detected by TPEF simultaneously with THG. We found that THG signals and TPEF fluorescence co-localized in fixed and stained animals, indicating that strong THG occurs at intestinal lipid droplets (**Figure 2b–d**). Minimal or no changes in the intensity or the localization of THG signals collected from intestinal areas was observed in animals stained with these dyes.

**Figure 3:**
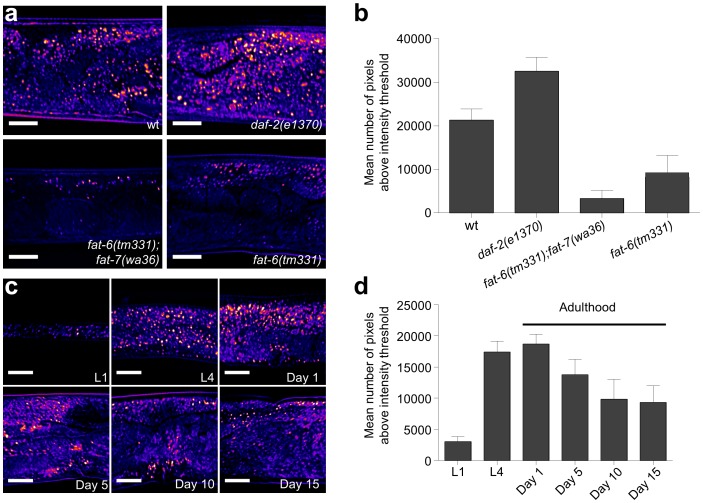
THG imaging of lipid droplets in the intestine of wild type worms and mutant animals with altered lipid storage profiles. (**a**, **b**) Intestinal lipid content of wild type animals compared with *daf-2, fat-6;fat-7 double,* and *fat-*6 single mutants. Quantification of THG signals is shown in panel b (n = 10 for each mutant, *P*<0.001, one-way ANOVA followed by Tukey HSD *post hoc* test, error bars denote S.E.M, scale bars in images denote 20 µm). (**c**, **d**) THG imaging of lipid content at specific developmental times (L1 and L4 larval stages) and during ageing (at day 1, 5, 10 and 15 of adulthood). Quantification of THG signals is shown in panel d (n = 10 for each time point, error bars denote S.E.M, scale bars in images denote 20 µm).

We further assessed the performance of THG in detecting lipid depositions by imaging *C. elegans* mutants with altered fat storage profiles. Mutations in the *daf-2* gene encoding the nematode insulin/IGF receptor cause increased lipid deposition compared to wild type worms [18]. By contrast, lesions in the Δ9 fatty acid desaturases FAT-6 and FAT-7 diminish intestinal fat content [19], [20]. THG imaging confirmed previously reported, dye-based and label-free CARS/SRS observations relevant to fat storage in these mutants (**Figure 3a, b**). Finally, we analyzed lipid deposition during *C. elegans* larval development and in adult animals during ageing. Consistent with previous studies [14], we found that fat content markedly increases during development, followed by subsequent gradual reduction during adulthood (**Figure 3c, d**).

We have applied THG microscopy to assay various aspects of fat storage in a non-invasive and label-free manner, using live *C. elegans* animals. Our observations, in their totality, indicate that THG is a reliable, convenient and cost-effective method for imaging lipid depositions in biological samples. The exact sensitivity of THG in detecting lipids in various tissues and biological samples of different origin remains to be determined. Our observations in *C. elegans* indicate that THG provides signals of intensity that is comparable to that generated by two-photon excitation of lipophilic dyes, such as Bodipy and Nile Red (**Figure 2**). In this study, we focused on the intestine, primarily because it is the main lipid-storage site also the tissue that is most readily stained with conventional dyes in *C. elegans*, Nevertheless, we anticipate tha THG will also be usable in visualizing ectopic accumulation of lipids in non-adipose tissues that has been associated with pathology in humans (lipotoxicity).

Because it requires only one pulsed laser light source, THG can be readily implemented by appropriately modifying a common multiphoton confocal microscope, thus, significantly reducing the complexity of the setup, and facilitating pairing with microfluidics devices for high-throughput or longitudinal studies. THG signals can be converted to a binary image for the measurement of number, size and distribution of lipid droplets using particle analysis functions of imaging software such as ImageJ. This approach provides a robust means for lipid droplet analysis. Moreover, coupling of both THG and TPEF modalities in a single microscope allows the simultaneous visualization of non-fluorescent neutral lipids and autofluorescent deposits or aggregates such as lipofuscins. Thus, THG microscopy is a versatile and easily accessible tool that should facilitate the investigation of cellular and molecular mechanisms controlling fat metabolism and storage.

## Materials and Methods

### Nonlinear Microscopy Setup

A femtosecond t-Pulse laser (Amplitude Systems, France) was used for sample imaging (1028 nm, 50 MHz, 1 W, 200 fs). The average laser power on the specimens during experiments was 16 mW (0.32 nJ per pulse). Adjustable neutral density filters (New Focus, Newport Corp., USA) were used to precisely control the power at the sample plane. A set of galvanometric mirrors (Cambridge Technology, USA), for sample scanning was placed on a modified upright optical microscope (Nikon Eclipse; Nikon Corp., Japan). The focal plane was selected with a motorized translation stage (Standa Ltd., Lithuania; 1 µm minimum step). A telescope system, which expands the laser beam approximately six times, was used to fill the back aperture of the objective lens. The beam was tightly focused on the sample plane by using a high numerical aperture objective lens (Carl Zeiss, C-Achroplan 32×, N.A. 0.85, water immersion; Carl Zeiss GmbH, Germany). Specimens were placed between two very thin (∼70 µm) round glass slides (Marienfeld GmbH, Germany). Glass slides were separated with a 100 µm thick spacer in order to avoid damaging specimens. Sample scanning and data acquisition were controlled through a Lab View interface. A CCD camera (PixeLINK) was used for observation. The two non-linear signals (THG and TPEF) simultaneously generated at the focal plane were detected via different channels; one in transmission and the other in reflection mode. This is essential for performing co-localization experiments. TPEF signals were recorded via the reflection mode by using a photomultiplier tube (PMT; Hamamatsu), attached at the position of the microscope eyepiece and connected to a computer. A short pass filter (SPF 700 nm, CVI) was placed at the PMT input to cut off reflected laser light, and a color glass filter (LP 530 nm, CVI) eliminated reflected second harmonic signals. A condenser lens (40×, 0.75 NA, Carl Zeiss, Plan-Neofluar) was used for the collection of THG signals in transmission mode (for thin samples most of harmonics generation light propagates in the direction of the fundamental laser beam). After passing through a colored glass filter (U 340-Hoya), THG signals reached a second PMT tube (Hamamatsu) and were recorded simultaneously with TPEF signals. With this setup, a 600×600 pixel THG or TPEF scan is recorded in less than two seconds. To improve the signal to noise ratio, 20 scans are averaged for each final image (total scan time for each image is approximately 35 s). To further improve image quality, a series of 2D optical sections was acquired at 2 µm intervals (z stack) and projected (maximum intensity projection) onto a single plane. Image J (NIH, http://imagej.nih.gov/ij/) was used for viewing and processing image data.

### *C. elegans* Strains and Maintenance

We followed standard procedures for *C. elegans* maintenance. Briefly, worms were kept at 20°C and maintained on standard NGM plates seeded with *E. coli* OP50 bacteria unless otherwise noted. The strains used include Bristol N2: wild-type, CB1320: *daf-2(e1370)III*, BX106: *fat-6(tm331)IV*, and BX156: *fat-6(tm331)IV;fat-7(wa36)V*.

### Lipid Staining

NGM plates were seeded with OP50 *E.coli* bacteria and allowed to dry overnight at room temperature. The following day, bacteria were killed using 48 W UV 254 nm irradiation lamps for 10 min (Bio-Link BLX-E365, Vilber Lourmat, France). For Bodipy staining, 1 µM Bodipy 500/510 (Sigma-Aldrich Corp., USA), calculated to the volume of the plate, was added from a stock solution of 5 mM diluted in 100% DMSO, onto the plate surface and allowed to dry at room temperature. L4 N2 worms were incubated on these plates and transferred to fresh plates every other day until they reached day-4 of adulthood. For epifluorescence and THG imaging, worms were anesthetized with 10 mM sodium azide and mounted on glass slides. For Nile Red staining, L4-stage worms were incubated on plates seeded with UV-killed OP50 bacteria and transferred to fresh plates every other day until they reached day-4 of adulthood. Prior to viewing, worms were fixed for 5 min in 3 mL cold (−20°C) methanol. Subsequently, 2 mL of PBTw (PBS with 0.1% Tween-20) were added and tubes were centrifuged for 2 min at 3000 rpm to remove the supernatant. Finally, worms were washed twice in PBTw. Following fixation, worms were stained for 20 min in 10 µM Nile Red (Sigma-Aldrich Corp., USA), added from a stock solution of 50 mM Nile Red diluted in 100% DMSO. For Oil Red-O staining, a stock solution was prepared by adding 0.5 g Oil Red-O (Sigma-Aldrich Corp., USA), in 100 mL isopropanol and allowing it to slowly dissolve on the bench for several days. A new solution consisting of 40% stock solution and 60% distilled water was allowed to sit for 10 min and subsequently filtered with a 0.4 µm filter [1]. L4-stage animals were grown for specified time intervals on plates with UV-killed OP50 bacteria, then collected and fixed with methanol. For staining, worms were incubated in Oil Red-O filtered solution for 20 min.

### Image and Statistical Analysis

THG signal quantification was performed by setting a threshold in the obtained normalized slice images, so that regions generating high levels of nonlinear signal (mainly corresponding to lipid particles) are solely detected and isolated. Processing of images and thresholding was performed using Image J. Normalized 8 bit slice images of the sample are initially inserted in ImageJ and consequently thresholded using a constant threshold value so that only the highest 20% of the THG signals is recorded. In this manner, the generated stack of binary images following the thresholding procedure represents exclusively the lipid droplets in the intestine of the animal, while the lower THG signals arising from other inhomogeneous structures are effectively eliminated. Lipid content was measured by calculating the total area of detected regions for all sequential optical planes covering the sample depth. At least 10 animals were imaged for each genetic background or time point examined. Mean pixel intensities were calculated by averaging values obtained for each image, after thresholding, in Image J Detection and area measurement of lipid droplet regions in the resulting binary images stack was performed through the Analyze Particles function of ImageJ. The total sum of the detected areas (in pixels) is calculated as a representative index of the total lipid content within the examined part of the intestine. Total lipid particle area measurements of different samples were compared by one-way ANOVA, followed by Tukey HSD *post hoc* tests (SPSS, IBM Corp., USA).

## Supporting Information

File S1 **Optical Harmonics Generation by focused Gaussian beams.**
(DOCX)
