# Supplementary material for: Label-Free Imaging of Lipid Depositions in C. elegans Using Third-Harmonic Generation Microscopy
Source: PLoS One. 2014 Jan 2;9(1):e84431. doi: 10.1371/journal.pone.0084431 (PMC3879321; doi:10.1371/journal.pone.0084431)
Supplement: File S1 — Optical Harmonics Generation by focused Gaussian beams. (DOCX) [file pone.0084431.s001.docx]

**Optical Harmonics Generation by focused Gaussian beams.** Most of the practical applications of optical harmonics generation, including microscopy, presuppose the use of a tightly focused Gaussian laser beam, so that the efficiency of the non-linear process can be increased by several orders of magnitude in comparison to an unfocused excitation beam [[1](#_ENREF_1),[2](#_ENREF_2)]. The mathematical description for this situation is based on the solution of the non-linear optical wave equation

where $\vec{E}$ is the electric field of the wave, $n$ is the respective refractive index of the medium, $c$is the speed of light in vacuum, $\mu_{0}$ is the permeability of free space and $\vec{P}^{NL}$is the non-linear part of the total induced polarization. The analytical solution of the above equation for a focused Gaussian beam results in the following approximate expression for the amplitude of the N-th harmonic wave $A_{0}^{(N)}$

$$A_{0}^{(N)}=\frac{iN\omega}{2n_{N}c}\chi^{(N)}(A_{0}^{(1)})^{N}J_{N}(\Delta k,z_{0},z)$$

where $\chi^{(N)}$ is the N-th order non-linear susceptibility of the medium, $\omega$ is the angular frequency of the fundamental wave, $c$is the speed of light in vacuum, $n_{N}$is the refractive index experienced by the N-th harmonic wave and

where the z direction is specified as the dominant direction of wave propagation. The z_0_ constant represents the value of z at the entrance to the nonlinear medium, b is the confocal parameter of the fundamental beam, while $\Delta k$ stands for the wave vector mismatch between fundamental and N-th harmonic wave and is given by the expression

$\Delta k=Nk_{1}-k_{N}$.

$J_{N}$ can be calculated analytically for two instances: the first is the plane wave limit, where $b>>\left|z_{0}\right|,\left|z\right|$. In this case, the denominator of the above integral is approximately equal to unity, so that

or in an alternative form

$$\left|J_{N}(\Delta k,z_{0},z)\right|^{2}=L^{2}\sin c^{2}\left(\frac{\Delta kL}{2}\right)$$

where the interaction length L is given by the relation

$L=z-z_{0}$ .

The second, reverse limiting case is that in which the fundamental wave is tightly focused within the volume of the medium so that $b<<\left|z_{0}\right|,\left|z\right|$. For such a tightly focused beam, the limits of the initial integral can be replaced by plus and minus infinity

to derive a straightforward contour integration that

$$J_{N}(\Delta k,z_{0},z)=\left\{\begin{matrix} 0 ,\Delta k\leq0, \\ \frac{b}{2}\cdot\frac{2\pi}{(N-2)!}\left(\frac{b\Delta k}{2}\right)^{N-2}e^{-b\Delta k/2} , \Delta k>0. \end{matrix}\right.$$

Therefore, for N = 3 in the above expression, we find that THG vanishes when the perfect phase matching condition is met ($\Delta k=0$) and is maximized through the use of a positive mismatch value ($\Delta k>0$). This result can be explained through the Gouy phase shift by π radians, which is experienced by any focused light beam. For example, let us assume a focused Gaussian laser beam of angular frequency ω which interacts within a non-linear material of third order susceptibility χ^(3)^, where the perfect phase matching condition is fulfilled. Due to the π radians phase shift, the electric fields before and after the beam waist position will be described by exactly opposite functions and the same will be true for their respective third order nonlinear polarizations which depend on the cube of the incident field. This implies that the symmetrically generated third harmonic waves around the beam waist position will interfere totally destructively since the third order polarization vector $\vec{P}^{(3)}$ which accounts for THG follows the incident field $\vec{E}$ within the first half of the focal volume, while at the second half it shows exactly the opposite behavior. Finally, a negative value of $\Delta k$ is not able to induce efficient generation of third harmonic signal, due to the momentum conservation restriction of the involved photons.

**References**

1. Boyd RW (2008) Nonlinear Optics. Burlington, MA: Academic Press.

2. Masters BR, So PTC (2008) Handbook of Biomedical Nonlinear Optical Microscopy. New York: Oxford Univ. Press.
